# Gene conservation of six Hungarian local chicken breeds maintained in small populations over time

**DOI:** 10.1371/journal.pone.0238849

**Published:** 2020-09-08

**Authors:** Nora Palinkas-Bodzsar, Nikoletta Sztan, Tamas Molnar, Andras Hidas

**Affiliations:** 1 National Centre for Biodiversity and Gene Conservation, Institute for Farm Animal Gene Conservation, Godollo, Hungary; 2 Kaposvar University, Kaposvar, Hungary; 3 Szent Istvan University, Godollo, Hungary; National Cheng Kung University, TAIWAN

## Abstract

Investigation into the genetic diversity of certain endangered native breeds of domestic animals has been in common practice for several decades. The primary objective of these investigations has been to reveal the exceptional genetic value of such breeds, both for their conservation and also to gain insight into their current genetic status, as they have been undergoing a progressive decrease in population size and general diversity; this has been compounded by the general lack of an optimal breeding scheme. In this study, we have investigated changes in the genetic diversity of six Hungarian local chicken breeds based on 29 microsatellite loci over a period of 15 years. In terms of the basic diversity measures, populations sampled in 2017 generally exhibited a lower heterozygosity and mean number of alleles and thus, experienced a higher degree of inbreeding. Although the effective population size increased, the estimates of populations sampled over different periods indicated comparatively low values, suggesting overall lower genetic variance. Pairwise F_ST_ estimates were higher in the populations sampled in 2017, showing a larger genetic distance between them. Considerable differences exist between the populations of the same breeds, which can most likely be attributed to genetic drift. STRUCTURE results have shown a clear separation between the Hungarian populations, which is in agreement with the principal coordinate analysis. The most likely clustering was found at K = 6, classifying the populations of the same breed as one group. No considerable allele loss was found in the Hungarian indigenous chicken breeds after 15 years of conservation. In general terms, after 15 years, the level of inbreeding within the populations was, in fact, higher, although this could be effectively reduced through the use of an improved mating system. Consequently, the breed management applied in the case of Hungarian local chicken breeds was found to be effective at adequately conserving their genetic variability.

## Introduction

Over the last several decades, exploration has been under way regarding the genetic diversity of endangered domestic animal breeds. The loss of genetic variability, together with the limited number of commercial hybrids, both suggest that genetic diversity in poultry is one of the most endangered genetic resources [1–3]. There are numerous grounds supporting gene conservation in indigenous breeds of chicken. From the point of view of conservation, it is necessary for native chickens to be preserved as a genetic resource, revealing their particular and distinctive genetic value for the purpose of future breeding. They are capable of providing a source for unique alleles and facilitate enrichment with genes associated with health and quality traits [4, 5]. Another justification for studies in this area is to carefully observe the genetic status of the populations themselves, as native chicken breeds in conservation breeding programmes are often maintained in small populations, and therefore more affected by loss of genetic variance and inbreeding depression. Local chicken flocks typically have no pedigree data, suffer from changing population sizes (bottlenecks), and lack properly planned breeding programmes [6]. It is clear that those unique combinations of genes which make up a specific genotype are at the risk of disappearance, in spite of the fact that they may constitute potentially beneficial traits [7].

Maintaining an effective population size (Ne) plays an important role in conservation genetics, as it is a determining factor in the development of the population in regard to inbreeding and genetic drift. Minimising loss of genetic variability is one of the main concerns of conservation breeding programmes and can be achieved through the maximisation of the effective population size, and hence, decrease the occurrence of genetic drift. Estimation of the effective size of a population and the monitoring of its changes over time are important to accurately assess the effectiveness of the breed management, particularly in small populations, where the allele frequencies change more easily over time. In the absence of selection, any change in allele frequency could solely be the result of drift and thus indicates the effective size of the population [8].

Although high-throughput SNP techniques have been more preferred for a while [9], microsatellite markers have similarly proven to be efficient molecular tools particularly in temporal studies [10] for assessing changes in genetic diversity as well as for setting priorities for conservation [11]; this has been especially applicable in the case of local chicken breeds [12–18]. Nevertheless, despite the high level of thoroughness and attention to detail in the investigations, any results obtained can easily become obsolete in the case of small populations where lifespans are more limited, and thus generations tend to change more frequently. Genetic diversity within these small populations is slowly, but steadily, eroded by the limited number of offspring (retained from the parents), especially in the absence of a specialised breeding scheme [19]. This is mostly the case, since it can be generally observed that the costs of animal breeding are constantly increasing and its income potential is decreasing, owing to the aggressive development and competition from intensive production practices [20].

Having a long history in this area, one of the primary activities of the National Centre for Biodiversity and Gene Conservation (Godollo, Hungary) has been the preservation of local Hungarian poultry species. The aim of this study was to investigate the extent of divergence which has developed in the genetic diversity of six Hungarian indigenous chicken breeds over a period of 15 years. We trust that our results will provide useful information regarding small populations which have been managed for the purpose of maintaining their genetic variation in gene banks for gene conservation as well as future breeding programmes.

## Materials and methods

Animals were kept and maintained according to general animal welfare prescriptions of the Hungarian Animal Protection Law (1998; XXVIII). Permission to undertake experimental animal research at the National Centre for Biodiversity and Gene Conservation was granted by the National Food Chain Safety Office, Animal Health and Animal Welfare Directorate, Budapest (Permission no. PE/EA/2485-6/2016). All experimental methods described herein were approved by the Institutional Ethics Review Board.

### Sample collection and DNA extraction

Six native Hungarian chicken breeds were included in this study: the Yellow (HY), the Speckled (HS) and the White Hungarian (HW), as well as the Speckled (TNNS), the White (TNNW) and the Black Transylvanian Naked Neck (TNNB). A major breeding program has resulted in good dual-purpose, medium-sized Hungarian chicken breeds which have been propagated not only for their sizable egg production (140–150 pieces per year per hen, egg weight: 50–55 g) but also for their excellent meat quality (fine-fibred, highly-palatable). In the case of the HY, HS and HW breeds, the body weight of the hens is 2.0–2.3 kg, while that of the roosters is typically 2.5–3.0 kg. The Transylvanian Naked Neck chickens are characterized by their featherless necks and only slight plumage on the top of their heads. These breeds are extraordinarily resistant and known for their good winter-laying ability; however, their body weight (hens: 1.7–2.2 kg, roosters: 2.2–2.4 kg) and egg production (80–100 pieces per year per hen, 48–50 g) are slightly lower when compared to the feathered Hungarian breeds [21]. 1 ml of individual blood samples were collected from each breed following the 15 year period, from thirty birds per population (20 females and 10 males), and sodium citrate solution was used as anticoagulant. Overall, 180 DNA samples were extracted using the traditional salting-out method [22], modified for chickens [23]. DNA concentration of the samples was measured using a NanoDrop spectrophotometer (Thermo Fisher Scientific, USA), then equalized to 5 ng/μl. The samples were stored at –20°C until use.

### Microsatellite genotyping

Individuals were genotyped using the same set of 29 microsatellite loci recommended by the FAO [24], as in our previous study [23], to obtain information as to how the genetic diversity of Hungarian indigenous chicken breeds has changed over a period of 15 years. To reduce the costs of the study, tailed primers were used for genotyping; therefore, instead of applying directly-labelled primers, a universal sequence (5'-CAGGACCAGGCTACCGTG-3') was placed before the 5’ end of the forward primers [25]. This universal primer (tail) was labelled with varying fluorescent WELL-RED dyes (D2, D3, D4); and thus–with proper optimization–the markers could be grouped into 10 multiplex PCR reactions, taking into consideration the fragment sizes, and the PCR products were pooled according to the fluorescent colours for the electrophoretic analysis (S1 Table).

The final volume of 15 μL master mix contained 5 μM primer, 10x Dream Taq Buffer with 20 mM MgCl_2_ (Thermo Scientific), 25 mM dNTP mix (Thermo Scientific), 5 U/μL Taq DNA polymerase (Dream Taq DNA polymerase, Thermo Scientific) and 25 ng genomic DNA. The ratio of the tailed forward and reverse primers and its fluorescently-labelled tail sequence was 1:2:1. PCR profile: 95°C for 4 min denaturation followed by 30 cycles of amplification: 95°C for 15 sec, 60°C for 30 sec and 72°C for 1 min, with a final extension at 72°C for 9 min (Kyratec Trinity Supercycler). The annealing temperature and the number of cycles were optimized for each multiplex reaction respectively (S1 Table).

PCR products were detected by capillary gel electrophoresis using an automatic DNA sequencer (GenomeLab ™ GeXP Genetic Analysis System, USA) according to the manufacturer’s instructions. A 400 bp allele ladder (400 bp size standard) was used to determine the allele sizes. For each population, 5 reference samples were selected from our earlier study [23] and genotyped with the tailed primer sets. The results were evaluated per sample using the GenomeLab Genetic Analysis System software (Beckman Coulter). Data correction was performed on the basis of the previously-obtained allele frequencies [23] and the allele sizes of the reference samples.

### Genetic diversity estimates within and between populations

Allele frequencies, mean number of alleles (MNA), and expected and observed heterozygosity (H_E_ and H_O_) were estimated across all loci in each population by the use of an Excel Microsatellite Toolkit [26]. Effective population sizes (Ne) were calculated using the linkage disequilibrium (LD) method (minimum allele frequency: 0.01) [27] for each population as well as the Jordan/Ryman temporal method [28] for generations as they are implemented in NeEstimator v2.1 [29]. Estimating the effective population size based on gametic disequilibrium, has the advantage that it only requires a single sample from the population. The LD method has greater significance when the effective size of the population is small and may therefore be of considerable use to conservation biologists, who are often faced with low or potentially low Ne [30]. In general, small population sizes are more expected to give rise to relatively-high levels of LD, and conversely, large population sizes to low LD levels. A noteworthy feature of this means of estimation is that a measurement taking at a single, given point in time is suitable for providing a reliable assessment of effective size. In the field of conservation, it is usually the most-recent population sizes that are of greatest interest, and for which unlinked loci are the most relevant [31]. The temporal method utilizes data on the rate of change in allele frequencies between samples taken at different points in time and calculates an average estimate of genetic drift over the loci [28]. The inbreeding coefficients (F_IS_) of each Hungarian population, including the test for significance and Weir & Cockerham [32] estimates of Wright’s fixation indices (F_IT_, F_ST_, F_IS_), were calculated with FSTAT software [33]. Values of standard error were generated by jack-knifing over both loci and the populations. A molecular analysis of variance (AMOVA) was calculated using GenAlEx software [34, 35], which partitions genetic variability into separate components. A principal component analysis (PCA) by the varimax rotation method was performed on the basis of diversity estimates (MNA, H_E_, H_O_, F_IS_) using SPSS 11.5 software [36]. Pairwise F_ST_ values were computed with GENEPOP software [37].

### Cluster analysis

Clustering of individuals from multi-locus genotypes was performed using a Bayesian-based approach with the STRUCTURE software [38]. The analysis was performed using an admixture model with correlated allele frequencies and run with 20,000 burn-in periods, followed by 50,000 iterations for each K number ranging from 2 to 12. For each K value, 100 independent runs were performed. Pairwise comparisons of the 100 solutions for each K value were made in a greedy algorithm, and clusters with the highest average similarity index were visualized using the CLUMPAK software package, which provided a summary and graphical representation of the STRUCTURE results [39]. The most likely clustering (ΔK) was estimated by STRUCTURE HARVESTER web v0.6.94 [40] using Evanno’s method [41]. In contrast, a principal coordinate analysis (PCoA) via a covariance genetic distance matrix with data standardization was conducted with GenAlEx software 6.5 [34, 35] and a two-step cluster analysis was performed on the individual diversity scores (originating from the PCA) using the SPSS 11.5 software [36]. PCA creates a clustering of the individuals based on their diversity scores and the direction of the changes observed can be easily visualized as the two axes of the plot represent different meanings.

## Results

### Genetic diversity of Hungarian chicken populations after 15 years of conservation breeding

The diversity of six Hungarian chicken breeds was analysed in relation to a new set of the same breeds sampled following a period of 15 years.

In total, 157 alleles were found in those Hungarian populations which were sampled in 2002 across all 29 microsatellite loci, while 152 alleles were obtained in the populations sampled in 2017. The mean number of alleles per population ranged from 2.9 (TNNW_2002_, TNNB_2002_) to 3.9 (HW_2002_) in the stocks sampled in 2002, while, in the populations sampled after a period of 15 years, this value ranged from 3.1 (TNNW_2017_) to 3.4 (HS_2017_) (Table 1). The heterozygosity observed in the populations sampled in 2002 did not differ significantly from the frequencies which were expected (except for HW_2002_, *P<0.05) inasmuch as the populations were in Hardy-Weinberg equilibrium. This is indicated by F_IS_ values, as opposed to the populations which were sampled in 2017, where the heterozygosity estimates of only two stocks (HY_2017_, HS_2017_) were significantly different from zero at P<0.05 (Table 1). Effective population size (Ne) estimates for the linkage disequilibrium (LD) method ranged from 15.6 (HW_2002_) to 27.3 (HS_2002_) in those populations sampled in 2002, while 15 years later, the point estimate range was 30.1–399.9 (HS_2017_–HY_2017_). When using the Jorde/Ryman two-sample (temporal) method, the lowest value was 4.4 (3.1–5.9) in the TNNS, while the highest was 12.7 (8.8–17.3) in the HY breed (Table 1).

**Table 1:** Mean number of alleles (MNA), expected (H_E_) and observed (H_O_) heterozygosity with standard deviation (SD), inbreeding coefficient (F_IS_), Hardy-Weinberg test (HW) and the effective population size (Ne) with 95% confidence interval (CI) in the Hungarian chicken populations.

Population	MNA±SD	H_E_±SD	H_O_±SD	F_IS_	HW	Ne (CI)^1^	Ne (CI)^2^
HY_2002_	3.1 ± 1.16	0.50 ± 0.036	0.51 ± 0.017	–0.021	ns	21.6 (16.7–28.9)	12.7 (8.8–17.3)
HY_2017_	3.2 ± 1.21	0.49 ± 0.035	0.48 ± 0.017	0.024	ns	399.9 (93.0–∞)	
HS_2002_	3.8 ± 1.21	0.55 ± 0.033	0.54 ± 0.017	0.031	ns	27.3 (21.9–34.8)	9.0 (6.5–11.9)
HS_2017_	3.4 ± 0.95	0.51 ± 0.036	0.50 ± 0.017	0.014	ns	30.1 (23–41.5)	
HW_2002_	3.9 ± 1.44	0.54 ± 0.033	0.51 ± 0.017	0.051	*	15.6 (13.2–18.5)	6.0 (4.3–7.9)
HW_2017_	3.3 ± 1.42	0.47 ± 0.038	0.43 ± 0.017	0.087	***	345.6 (93.2–∞)	
TNNS_2002_	3.2 ± 1.18	0.54 ± 0.029	0.53 ± 0.017	0.016	ns	23.4 (18.2–31.3)	4.4 (3.1–5.9)
TNNS_2017_	3.3 ± 1.42	0.56 ± 0.030	0.51 ± 0.017	0.082	***	55.5 (36.5–103.0)	
TNNW_2002_	2.9 ± 0.84	0.49 ± 0.032	0.51 ± 0.017	–0.039	ns	20.2 (15.4–27.3)	6.6 (4.5–9.0)
TNNW_2017_	3.1 ± 0.92	0.47 ± 0.032	0.42 ± 0.017	0.100	***	52.3 (33.6–102.0)	
TNNB_2002_	2.9 ± 0.96	0.44 ± 0.033	0.44 ± 0.017	0.015	ns	19.8 (15.1–26.6)	4.7 (3.2–6.3)
TNNB_2017_	3.3 ± 1.11	0.50 ± 0.034	0.45 ± 0.017	0.100	***	97.1 (53.0–375.5)	

HY = Yellow Hungarian, HS = Speckled Hungarian, HW = White Hungarian, TNNS = Speckled Transylvanian Naked Neck, TNNW = White Transylvanian Naked Neck, TNNB = Black Transylvanian Naked Neck.

Numbers in lower index of the populations indicate the year of sampling.

*P<0.05

***P<0.001, ns = non-significant.

^1^effective population sizes for each stock respectively, using the linkage disequilibrium method.

^2^effective population sizes for each breed by Jorde/Ryman two-sample (temporal) method.

**Fig 1:**
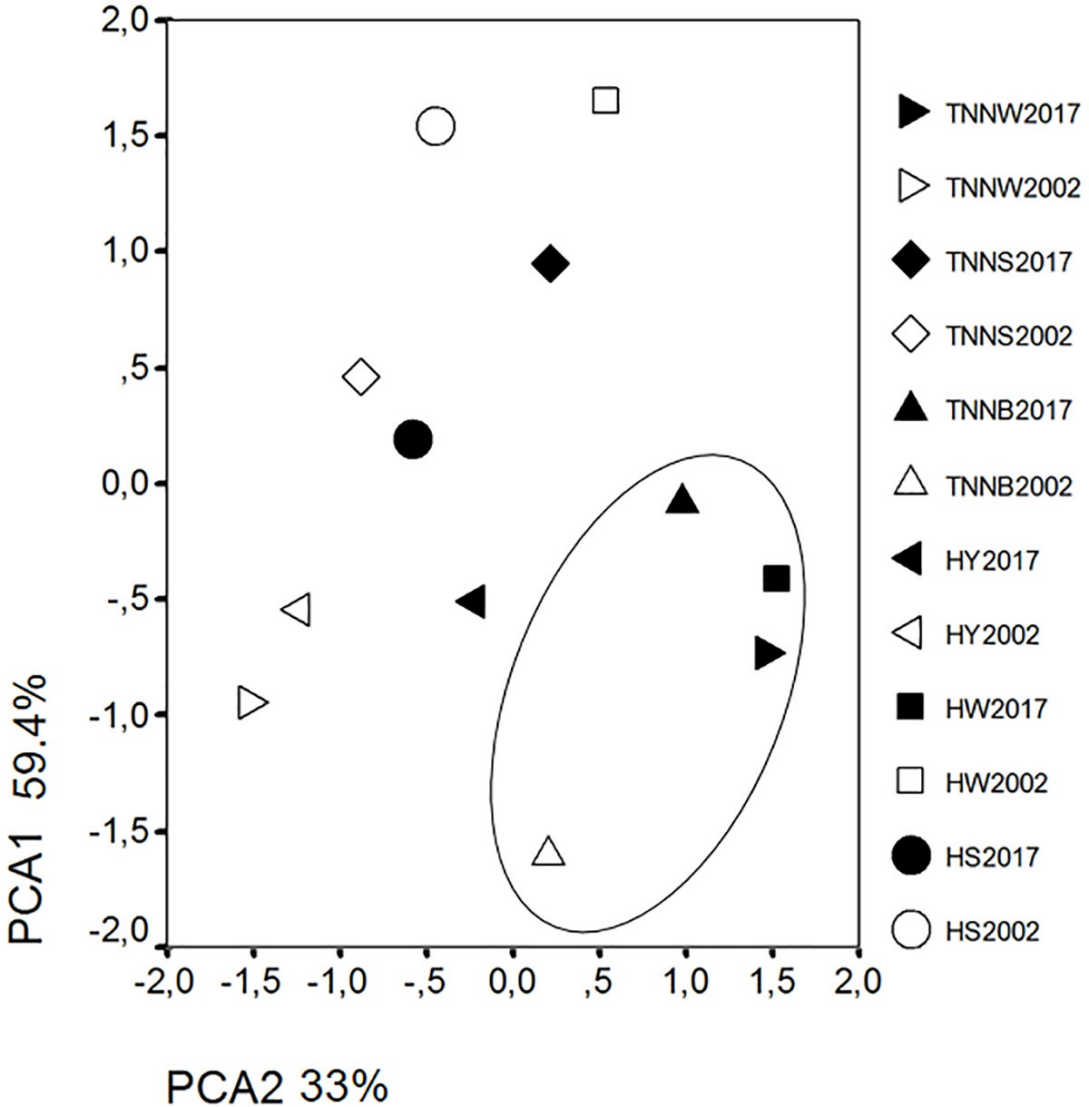
Principal component analysis of the Hungarian native chicken populations based on diversity estimates. Populations marked with an ellipse represent a separate cluster. TNNW = White Transylvanian Naked Neck, TNNS = Speckled Transylvanian Naked Neck, TNNB = Black Transylvanian Naked Neck, HY = Yellow Hungarian, HW = White Hungarian, HS = Speckled Hungarian. Numbers following the populations indicate the year of sampling.

A principal component analysis (PCA) of the summary diversity statistics was performed to collapse several parameters into two principal components (Fig 1). The first principal component differentiates populations mainly according to their mean number of alleles and expected heterozygosity [PCA1 = (0.504* MNA) + (0.221* H_O_) + (0.425* H_E_) + (0.200*F_IS_)], and the second, mainly by their inbreeding coefficients and observed heterozygosity [PCA2 = (0.279* MNA)–(0.395* H_O_)–(0.067* H_E_) + (0.645*F_IS_)]. Two principal components were extracted from the Hungarian native chicken populations with eigenvalues of 2.376 for the first principal component (PCA1), and 1.321 for the second (PCA2); these two principal components explained 92.4% of the total variance. Based on the cluster analysis of the individual principal scores of diversity the TNNB_2002_ and TNNB_2017_ stocks as well as the populations of the HW and TNNW breeds sampled in 2017 formed a cluster separate from the others, showing a higher level of inbreeding (Fig 1).

Wright’s fixation indices (average F_IT_, F_ST_ and F_IS_) of the Hungarian chicken populations are shown in Table 2. The inbreeding coefficient of the stocks sampled in 2002 at the total sample level (F_IT_) amounted to 0.241 ± 0.022. Its estimate was higher in those populations (0.298 ± 0.025) sampled 15 years later. The genetic differentiation (F_ST_) of the set from 2002 was slightly lower than the corresponding value of the populations sampled in 2017. Estimate of within (F_IS_) population variation was significantly lower in the stocks sampled in 2002 than those taken 15 years later. The results of AMOVA indicated the same trends (S2 Table).

**Table 2:** Wright’s F statistics of the Hungarian chicken populations on the basis of Weir & Cockerham (1984) estimations with standard deviations.

Year of sampling	F_IT_ ± SD	F_ST_ ± SD	F_IS_ ± SD
2002	0.241*** ± 0.022	0.233*** ± 0.014	0.010 ± 0.020
2017	0.298*** ± 0.025	0.248*** ± 0.009	0.066*** ± 0.032

Significantly different from zero at ***P<0.001.

Inbreeding coefficients: F_IT_ = overall population, F_ST_ = between population, F_IS_ = within population.

Pairwise F_ST_ estimates between the Hungarian populations sampled in 2002 varied between 0.15 (HW_2002_–TNNW_2002_) and 0.28 (HY_2002_–TNNB_2002_), while in case of the populations sampled after a period of 15 years the range was from 0.21 (TNNS_2017_–HY_2017_, TNNS_2017_–TNNB_2017_) to 0.30 (HW_2017_–HY_2017_). Values of the two populations of the same breed sampled at different points in time varied between 0.03 (HY_2002_–HY_2017_) and 0.08 (TNNS_2002_–TNNS_2017_, TNNB_2002_–TNNB_2017_) which are relatively low but still indicate a differentiation between them (Table 3).

**Table 3:** Pairwise F_ST_ estimates between the Hungarian local chicken populations.

Breed	HY _2002_	HY _2017_	HS _2002_	HS _2017_	HW _2002_	HW _2017_	TNNS _2002_	TNNS _2017_	TNNW _2002_	TNNW _2017_	TNNB _2002_	TNNB _2017_
HY_2002_												
HY_2017_	0.03											
HS_2002_	0.22	0.22										
HS_2017_	0.24	0.24	0.04									
HW_2002_	0.25	0.26	0.17	0.21								
HW_2017_	0.29	0.30	0.22	0.27	0.06							
TNNS_2002_	0.27	0.26	0.21	0.24	0.21	0.27						
TNNS_2017_	0.22	0.21	0.20	0.22	0.19	0.24	0.08					
TNNW_2002_	0.26	0.25	0.24	0.24	0.15	0.23	0.26	0.24				
TNNW_2017_	0.26	0.24	0.25	0.25	0.19	0.27	0.30	0.26	0.05			
TNNB_2002_	0.28	0.27	0.21	0.25	0.27	0.31	0.23	0.26	0.26	0.27		
TNNB_2017_	0.26	0.25	0.20	0.25	0.22	0.26	0.19	0.21	0.23	0.24	0.08	

Numbers marked with grey colour show the pairwise F_ST_ values between the populations of the same Hungarian breed sampled in 2002 and 2017.

HY = Yellow Hungarian, HS = Speckled Hungarian, HW = White Hungarian, TNNS = Speckled Transylvanian Naked Neck, TNNW = White Transylvanian Naked Neck, TNNB = Black Transylvanian Naked Neck.

Numbers in lower index of the populations indicate the year of sampling.

**Fig 2:**
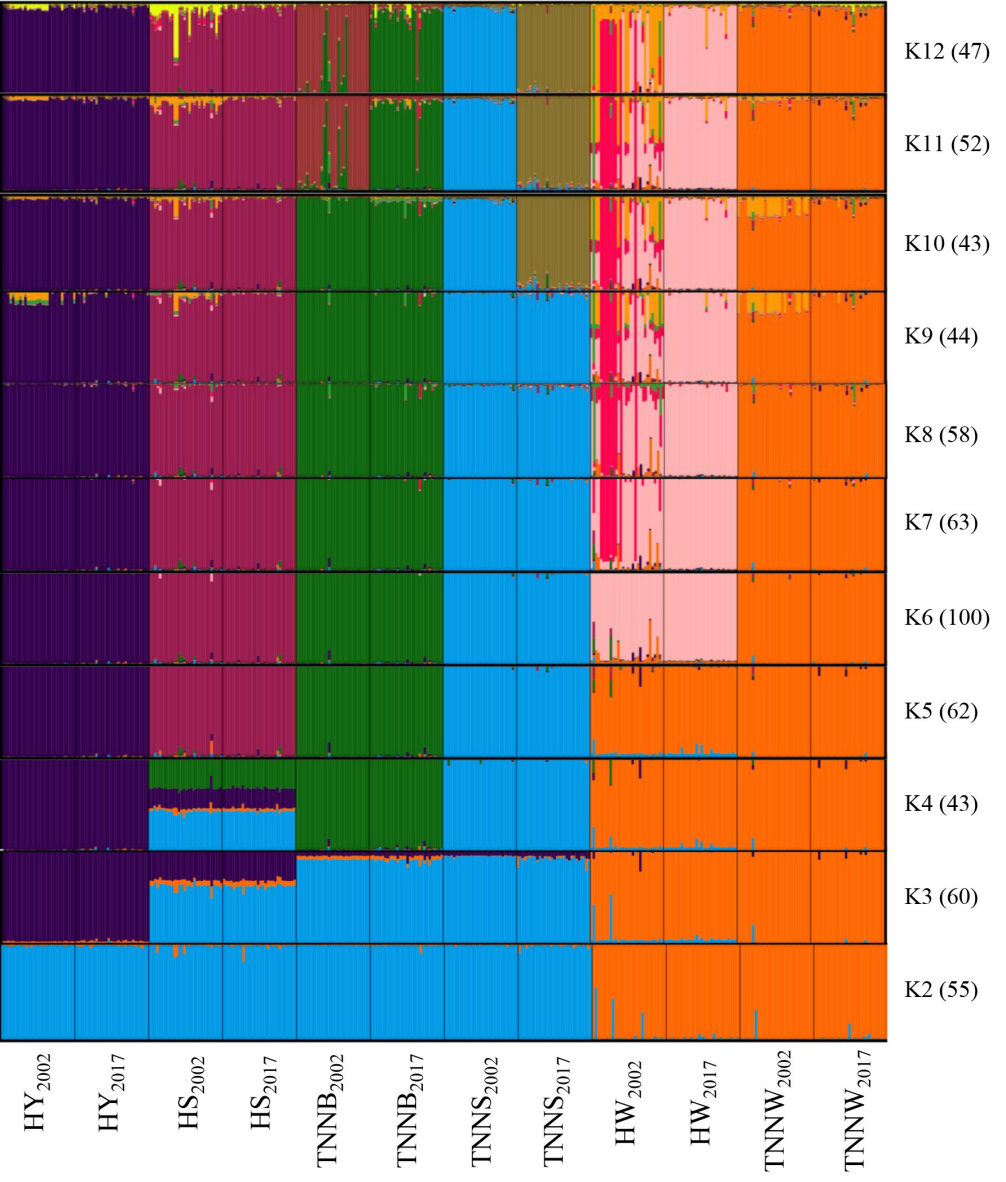
STRUCTURE clustering of the Hungarian native chicken populations. Numbers in parentheses indicate the identical solutions of the 100 runs at 95% threshold. HY = Yellow Hungarian, HS = Speckled Hungarian, TNNB = Black Transylvanian Naked Neck, TNNS = Speckled Transylvanian Naked Neck, HW = White Hungarian, TNNW = White Transylvanian Naked Neck. Numbers in lower index of the populations indicate the year of sampling.

The results of the STRUCTURE analysis are given in Fig 2. Due to the fact that this software places individuals into clusters without any previous information, all the 12 populations were analysed together for the purpose of acquiring reliable information on how the populations differentiated after 15 years of conservation. At the lowest K value (K = 2), populations of the HW and TNNW breeds diverged from the others and clustered together until K = 5. At K = 3, the HY_2002_ and HY_2017_ stocks formed a cluster and remained together until K = 12. The Transylvanian Naked Neck breeds (except for the TNNW) grouped with the HS stocks, which are slightly overlapping with the HY breed. At K = 4, the two TNNB populations split to form a separate cluster. The HS stocks are still overlapping with the HY and TNNB breeds, but at K = 5 they showed a clear population structure. The clustering of highest probability was obtained at K = 6 (N = 100) based on Evanno’s method, where the HW and the TNNW breeds separated from each other and this state remained until K = 12. From K = 6 to K = 9 the clustering was the same, however, the HW_2002_ showed a slightly-mixed population structure from K = 7. At K = 10 the two populations of the TNNS breed formed separate clusters, while at K = 11 the TNNB_2002_ and TNNB_2017_ stocks split off as individual groups. K = 11 and K = 12 showed identical solutions, indicating that a maximum of eleven clusters illustrate the data.

**Fig 3:**
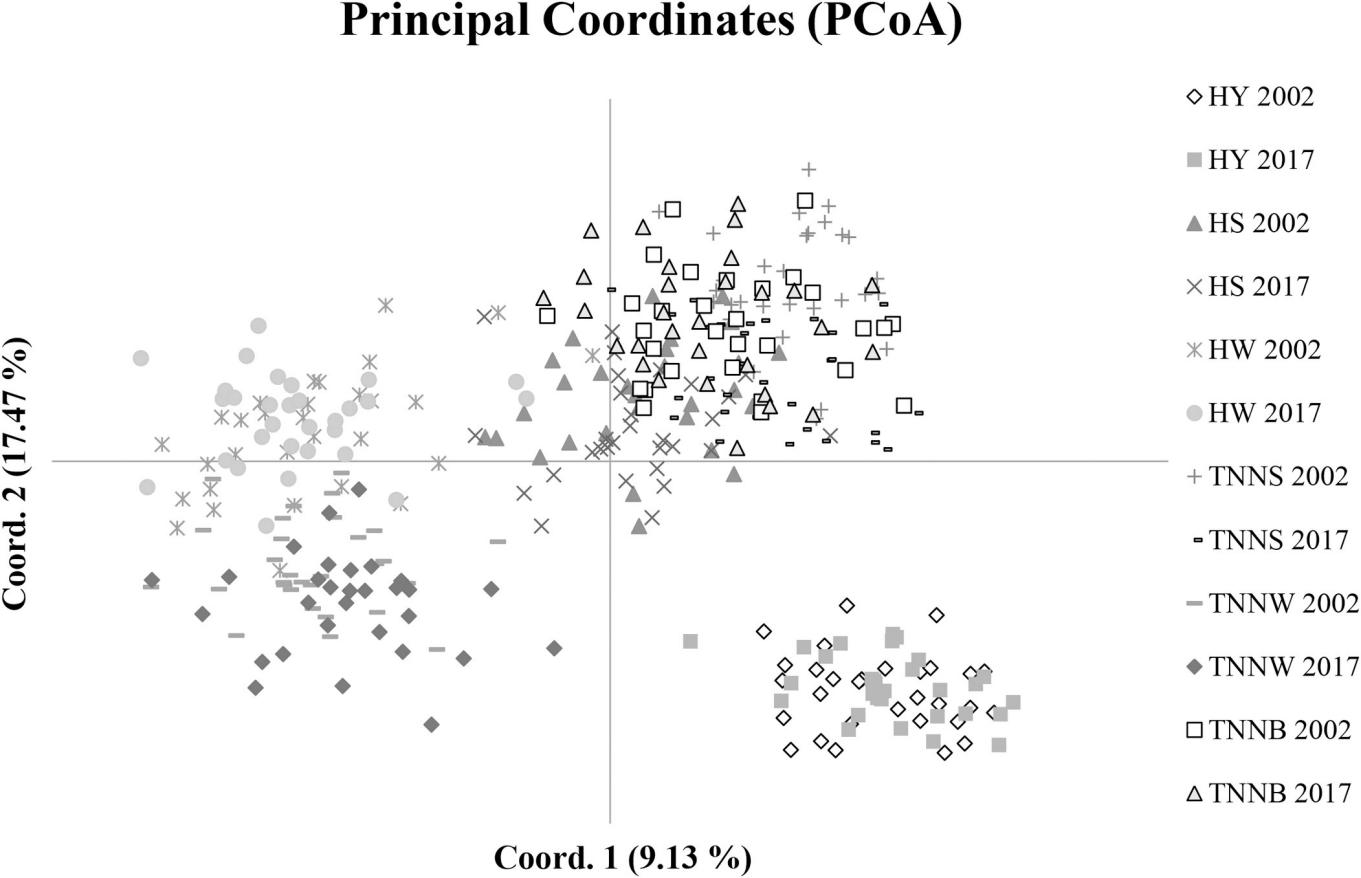
Principal coordinate analysis of the Hungarian native chicken populations via the covariance genetic distance matrix. HY = Yellow Hungarian, HS = Speckled Hungarian, HW = White Hungarian, TNNS = Speckled Transylvanian Naked Neck, TNNW = White Transylvanian Naked Neck, TNNB = Black Transylvanian Naked Neck. Numbers following the populations indicate the year of sampling.

Fig 3 illustrates the relationships of the Hungarian populations based on a principal coordinate analysis (PCoA) using individual multi-locus genotypes of 29 microsatellite markers. The percentage of variation explained by the first and second axes was 9.13% and 17.47%, with eigenvalues of 40.15 and 36.69, respectively. By the first component, the two populations of the HY breed clearly separated from the other groups. The left side of the diagram exhibits populations of the HW and TNNW breeds, showing a closer relationship between them. On the right side, above, the Transylvanian Naked Neck stocks (except for the TNNW) are grouped with the HS breed.

## Discussion

In the current study, we investigated the changes in the genetic diversity of six Hungarian local chicken populations based on genotyping individuals at 29 microsatellite loci over a period of 15 years. The observed heterozygosity values were decidedly similar to the expected ones, and F_IS_ estimates were not significantly different from zero, suggesting that the populations sampled in 2002 were close to the Hardy-Weinberg equilibrium state. After 15 years of conservation, heterozygosity estimates differed significantly in most of the breeds, consequently, the inbreeding coefficient (F_IS_) increased in all breeds with the exception of the HS. This is in agreement with the principal component analysis based on diversity estimates (Fig 3). Our basic diversity measurements, obtained after a period of 15 years, maintained their similarity to those estimated for 65 chicken populations at the same 29 microsatellite loci [42], and comparable to those reported by Zanetti et al. [15] for chickens native to Italy; however, authors of the latter study found a decrease of inbreeding coefficient for three out of five Italian populations after only 4 years of conservation [10]. Although the effective population size increased over the 15 year period, the Ne estimates of populations sampled at different points in time indicated quite low values, suggesting less genetic variance in those populations which were sampled 15 years later, due to the higher level of inbreeding (Table 1). The two methods used in our study for the purpose of estimating effective population size make calculations on different platforms: on the basis of linkage disequilibrium (LD method) and based on genetic drift (temporal method). The LD value is known to be less affected by drift compared to other factors. Accordingly, the value obtained by the LD method was higher, due to the fact that the effect of the factors influencing the LD value within a stock (e.g. population fragmentation, exchange of individuals within population, inbreeding) was smaller, owing to the appropriate breeding system. However, genetic drift did also occur over the 15 year period, as the population sizes were limited due to financial constraints. Thus, all things considered, the effective population size is lower in the case of the temporal method. These results are consistent with the findings in 37 European local chicken populations based on Wright’s Ne calculation [19]. In that study, the HW, TNNW and TNNB breeds were found to be the most inbred among the Hungarian populations, which was shown in our principal component analysis (Fig 1) as well.

The overall population inbreeding coefficient (F_IT_) indicated that heterozygote deficiency was lower in the Hungarian populations sampled in 2002 and it was almost fully explained by between-population differentiation (F_ST_, Table 2). In contrast, the populations, after 15 years of conservation, showed a higher F_IT_ estimate, suggesting the increased ratio of homozygote animals, this figure is still not as high as in the Swedish, Polish and Italian local chicken breeds [15, 43, 44]. The high F_ST_ values showed a clear population structure of both Hungarian sets, which is in agreement with the STRUCTURE results and notably similar to the findings in French native chicken breeds [45]. Compared to those Hungarian chicken populations which were sampled in 2002, those sampled in 2017 exhibited a higher degree of inbreeding (F_IS_); however, they were well managed in terms of maintaining the genetic variation found 15 years prior. The F_IS_ value of the Hungarian populations sampled in 2017 is even coherent with that of the Polish local breeds investigated [44]. After 15 years of conservation, the inbreeding level increased from 1% to 6% (F_IS_, Table 2), and the between-population differentiation changed from 23% to 25% (F_ST_, Table 2), which is consistent with the molecular analysis of variance (AMOVA, S2 Table). The reason for this might be attributed to the small population size itself (approx. 200 birds), as, in the cases of these populations the decreasing effects of genetic drift in the diversity may occur even more readily than allele losses.

The pairwise F_ST_ estimates observed between populations sampled in 2002 were generally a bit smaller than that 15 years later, indicating lower genetic similarity between the stocks sampled in 2017 (Table 3). The population differentiation was still not as high as local chicken breeds in Britain [16]. The difference had almost doubled between the HW and TNNW breeds by 2017 (from 0.15 to 0.27); however, in other cases, the similarity actually increased, mostly between the TNNS and HY breeds (from 0.27 to 0.21). The highest observed similarity between the two populations of the same breed was 0.08 (TNNS_2002_–TNNS_2017_ and TNNB_2002_–TNNB_2017_), which is remarkably close to the pairwise F_ST_ estimate (0.15) of two distinct breeds (HW_2002_–TNNW_2002_) sampled in the same year. This suggests that the diversity of TNNB and TNNS populations has changed more than that of the others. This concurs with the STRUCTURE results.

The STRUCTURE analysis (Fig 2) showed a clear clustering of the Hungarian local chickens. A similar population structure was observed for Italian native chicken populations even following a mere 4 years of conservation [10]. The results suggest that the two populations of the HY breed are indistinguishable, which is in concordance with the findings of the PCoA analysis based on genetic distances between individuals (Fig 3). Furthermore, the HW and TNNW breeds clustered together, similar to our previous study [23], but from K = 6 (most likely clustering), they were clearly separated, and their populations sampled in 2002 and 2017 remained together until K = 12, respectively, which was confirmed by the PCoA analysis (Fig 3). Individuals of the TNNB populations clustered with the HS and TNNS breeds at low K value, but constituted their own cluster at K = 5. Similar phylogenetic relationships were displayed by the PCoA analysis (Fig 3). The two stocks of this breed remained together up to a moderate level of resolution in the STRUCTURE analysis, but appeared as clearly distinguishable populations at K = 11. The two populations of the TNNS breed already appeared as separate groups at K = 10, suggesting a higher genetic differentiation between the stocks sampled at different points in time. This was confirmed by their relatively high pairwise F_ST_ estimate (Table 2).

The findings of the principal coordinate analysis are displayed in Fig 3 and show a strong concurrence with the STRUCTURE results. Individuals of the HY_2002_ and HY_2017_ populations formed a group distinct from the other breeds. The populations of the HS and TNNS breeds clustered with the TNNB flocks. Some birds of the HW_2002_ population bore similarity to the TNNS and TNNB breeds. This is also indicated by the STRUCTURE results at K levels higher than the most likely clustering; a slightly complicated population structure was discovered for the HW_2002_, which is comparable with the results obtained for an Italian local chicken breed [15]. Nevertheless, the majority of individuals from the HW_2002_ population were isolated from the other breeds on the left side of the graph; populations of the HW and TNNW breeds were almost completely differentiated.

In summary, no considerable allele loss was found in the Hungarian local chicken breeds after 15 years of conservation. However, one allele of the TNNB breed, having a frequency of 30%, disappeared; there was no significant change for the rest of the microsatellite loci. This may indicate a natural or artificial selection effect. It appears that there is a notable distance between the populations of the same breeds sampled at different points in time, especially between those of the TNNS_2002_-TNNS_2017_ and TNNB_2002_-TNNB_2017_ stocks, respectively, most likely due to genetic drift. This was also found in our previous study, which investigated subpopulations of the same breed maintained separately for 30–40 years as closed populations [23]. As for the HS and TNNS breeds, there were not enough eggs available in 2015 for the reproduction in our gene bank; therefore 300 eggs (nearly half of the hatched eggs) were taken from an elite breeder who had been maintaining these breeds for almost 20 years. This replacement had a remedial effect, especially for the HS breed, as its inbreeding coefficient not only did not deteriorate further, but rather displayed an improvement. The aforementioned facts may also have resulted in a slightly greater change in their genetic makeup.

The change of alleles between generations may be attributed to inbreeding, genetic drift, or even the sampling itself, due to the fact that these breeds are maintained in the gene bank having a relatively small population size (approx. 200 birds). Generally, the level of inbreeding appears to be higher after 15 years of conservation, but it is capable of being reduced through the use of a sire-rotation scheme different from that being used at present. The allele frequency of small populations may be subject to fluctuations by leaps, therefore, the allele set itself has proven to be a more stable indicator, as it changes only when the gene pool of the populations changes significantly, and this is not the case here.

The breed management which has been applied to these populations, i.e. maximising the genetic variance based on phenotypic traits and sire rotation used between families, has sufficiently demonstrated its suitability for adequately conserving genetic variability with the addition of some improvements. Thus, we hereby suggest the implementation of a controlled random male rotation scheme in place of the currently constant pattern of rooster exchange between families. Introducing new individuals into the breeding system from time to time may also help reduce the inbreeding level. Increasing the effective population size with either the use of a higher number of roosters in smaller families, or through the application of rooster exchange during periods of egg collection for pedigree, is recommended to conserve the genetic variance more effectively, however this practice does have its financial constraints.

## Supporting information

S1 Table Multiplex sets of the microsatellite markers used in this study. (DOCX)

S2 Table Summary of AMOVA results of the Hungarian chicken breeds. (DOCX)

S3 Table Microsatellite genotypes for the Hungarian chicken samples used in this study. (XLSX)
