# Supplementary material for: Gene conservation of six Hungarian local chicken breeds maintained in small populations over time
Source: PLoS One. 2020 Sep 8;15(9):e0238849. doi: 10.1371/journal.pone.0238849 (PMC7478839; doi:10.1371/journal.pone.0238849)
Supplement: S1 Table — (DOCX) [file pone.0238849.s001.docx]

**S1 Table: Multiplex sets of the microsatellite markers used in this study**

| **Fragment analysis** | **Multiplex PCR** | **Microsatellite markers** | **Allele sizes with tail sequence (bp)** | **Fluorescent labelling (WELL-RED)** | **Tm (°C)** | **Number of cycles** |
| --- | --- | --- | --- | --- | --- | --- |
| **FA1** | **M1** | **ADL0278** | 132-141 | D2 | 60 | 30 |
|  |  | **MCW0037** | 172-176 |  |  |  |
|  |  | **MCW0034** | 238-260 |  |  |  |
|  | **M6** | **MCW0078** | 153-161 | D4 | 56 | 30 |
|  |  | **MCW0014** | 182-196 |  |  |  |
|  |  | **LEI0166** | 374-384 |  |  |  |
| **FA2** | **M2** | **ADL0268** | 122-136 | D2 | 60 | 30 |
|  |  | **MCW0216** | 159-163 |  |  |  |
|  |  | **MCW0248** | 233-241 |  |  |  |
|  | **M7** | **ADL0112** | 142-150 | D4 | 58 | 35 |
|  |  | **MCW0222** | 238-244 |  |  |  |
|  |  | **MCW0098** | 281-283 |  |  |  |
| **FA3** | **M3** | **MCW0295** | 108-124 | D2 | 60 | 30 |
|  |  | **MCW0016** | 188-222 |  |  |  |
|  |  | **MCW0330** | 276-308 |  |  |  |
|  | **M8** | **MCW0081** | 132-153 | D4 | 56 | 35 |
|  |  | **MCW0069** | 176-194 |  |  |  |
|  |  | **LEI0094** | 267-301 |  |  |  |
| **FA4** | **M4** | **MCW0123** | 98-112 | D3 | 60 | 35 |
|  |  | **MCW0067** | 196-202 |  |  |  |
|  |  | **MCW0080** | 286-298 |  |  |  |
|  | **M9** | **MCW0165** | 132-136 | D4 | 62 | 30 |
|  |  | **MCW0104** | 208-244 |  |  |  |
|  |  | **MCW0103** | 284-288 |  |  |  |
| **FA5** | **M5** | **MCW0111** | 116-132 | D3 | 58 | 35 |
|  |  | **LEI0234** | 235-334 |  |  |  |
|  | **M10** | **MCW0020** | 197-215 | D4 | 60 | 35 |
|  |  | **MCW0206** | 241-257 |  |  |  |
|  |  | **MCW0183** | 314-340 |  |  |  |

The numerical order of the multiplex PCR reactions (M1-M10) are changed considering the fragment analysis
