# Supplementary material for: Gene conservation of six Hungarian local chicken breeds maintained in small populations over time
Source: PLoS One. 2020 Sep 8;15(9):e0238849. doi: 10.1371/journal.pone.0238849 (PMC7478839; doi:10.1371/journal.pone.0238849)
Supplement: S2 Table — (DOCX) [file pone.0238849.s002.docx]

**S2 Table: Summary of AMOVA results of the Hungarian chicken breeds**

| **Source** | **df** | **SS** | | **MS** | | **Est. Var.** | **%** | **Est. Var.** | **%** |
| --- | --- | --- | --- | --- | --- | --- | --- | --- | --- |
|  |  | **2002** | **2017** | **2002** | **2017** | **2002** | | **2017** | |
| **Among populations** | 5 | 714.81 | 748.12 | 142.96 | 149.62 | 2.26 | 23% | 2.36 | 25% |
| **Among individuals** | 174 | 1305.00 | 1364.87 | 7.50 | 7.84 | 0.08 | 1% | 0.56 | 6% |
| **Within individuals** | 180 | 1322.50 | 1209.00 | 7.35 | 6.72 | 7.35 | 76% | 6.72 | 70% |
| **Total** | 359 | 3342.31 | 3321.99 |  |  | 9.68 | 100% | 9.64 | 100% |

df = degree of freedom; SS = sum of squares; MS = mean squares; Est. Var. = estimated variance, given in percentage (%) as well
